# HIV-1 diversity in infected individuals in Suzhou and Suqian, China

**DOI:** 10.1186/s40064-016-2378-z

**Published:** 2016-06-24

**Authors:** Chenhao Qin, Ping Zhang, Weiguang Zhu, Fangyuan Hao, Aiping Gu, Ping Fen, Xueming Zhu, Hong Du

**Affiliations:** Department of Clinical Laboratory, The Second Affiliated Hospital of Soochow University, 1055 Sanxiang Road, Suzhou, 215004 Jiangsu China; Department of Clinical Laboratory, Suqian Center of Disease Control and Prevention, Suqian, Jiangsu China; Institute of Life Sciences, Jiangsu University, Zhenjiang, Jiangsu China

**Keywords:** HIV-1, Epidemiology, Recombination forms, Subtype distribution, CRF01_AE

## Abstract

Jiangsu is one province with severe HIV-1 epidemic in China. However, the molecular epidemiological characterizations of HIV-1 in many cities of Jiangsu remain unclear. A molecular epidemiological investigation was performed based on 38 HIV-positive samples collected from Suzhou and Suqian during 2011–2013. Five HIV-1 genomic fragments, *p17*, *pol*, *vif-vpr*, *vpr-env*, and *C2V3* were amplified and sequenced from these samples. HIV-1 group M subtype of each sample was determined by phylogenetic analyses with the standard reference sequences. Among these infected individuals, 81.6 % (31/38) self-reported to be infected via sexual contacts, including 50.0 % (19/38) via heterosexual contact and 31.6 % (12/38) via homosexual contact. Among 34 samples with available *pol* or *vif-env* sequence, 19 (55.9 %) CRF01_AE, 7 (20.6 %) CRF07_BC, 3 (8.8 %) CRF08_BC, and 5 (14.7 %) inter-subtype recombinants were identified. No pure B, B′ and C subtypes were found in this cohort. The five recombinants contain one B/C, three CRF01/B and one CRF01/B/C recombinants. These results suggest that CRF01_AE was the most predominant HIV-1 group M subtype and CRF01_AE-involved recombinants were the major recombinant forms. Comparison showed that there was no obvious difference in HIV-1 group M subtype distribution between Jiangsu (including Suzhou and Suqian) and the surrounding provinces (e.g., Shanghai, Anhui, and Shandong). CRF01_AE and CRF07_BC were the top two predominant HIV-1 genotypes in Jiangsu, and less and/or no pure subtype B and C was currently circulating here. We predicted that more CRF01/CRF07 recombinants, but fewer B/C recombinants will be generated in Jiangsu in future.

## Background

HIV/AIDS continues to be one of major public health issues in China. By the end of 2011, there were approximately 780,000 (620,000–940,000) persons living with HIV/AIDS (PLHIV) in China (Ministry of Health of the People’s Republic of China, Joint United Nations Programme on HIV/AIDS, World Health Organization 2012). The prevalence rates was 0.058 % (0.046–0.070 %) nationally. According to the case reporting system, all 31 provinces (including autonomous regions and municipalities) have reported HIV/AIDS cases. The numbers of infected cases were very different between provinces. The worst-hit provinces by HIV/AIDS in China are Yunnan, Xinjiang, Guangxi and Sichuan, accounting for 75.8 % of the national total (Ministry of Health of the People’s Republic of China, Joint United Nations Programme on HIV/AIDS, World Health Organization 2012; Li et al. 2013). Jiangsu, a province in the East China, which neighbors with Shanghai, Zhejiang, Anhui, Henan and Shandong, is one of regions with severe HIV-1 epidemic (Ministry of Health of the People’s Republic of China, Joint United Nations Programme on HIV/AIDS, World Health Organization 2012; Zhang et al. 2013; Guo et al. 2009a).

As one of the richest regions of China, Jiangsu is a typical labor force-import province and attracts a large number of migrant populations to work and live (Meng et al. 2011). The data in 2011 showed an obvious increase (17.3 %) in new HIV infections in Jiangsu compared to the data in 2010 (Control JPCfDPA 2012). About 72.0 % of PLHIV in Jiangsu were reported in five cities, Nanjing, Suzhou, Wuxi, Changzhou and Xuzhou (Control JPCfDPA 2012). Molecular epidemiological investigations based on *p17* and *C2V3* regions of HIV genome showed that majority of HIV-1 infection in Jiangsu were caused by HIV-1 group M CRF01_AE, B and C subtypes, and few were associated with HIV-1 recombinants (Control JPCfDPA 2012). The co-circulation of multiple HIV-1 group M subtypes in Jiangsu will provide more chances to generate new inter-subtype recombinants. As HIV-1 recombination often occurs in the *pol* and *vif*-*env* regions (Pang et al. 2012), some recombinants in Jiangsu might not be found by the analyses of *p17* and *C2V3* sequences. Recently, some new HIV-1 recombinants were reported in Jiangsu and several surrounding provinces, such as Anhui, Shanghai, Shandong (Wu et al. 2013; Zhong et al. 2007; Zhang et al. 2010; Guo et al. 2009b, 2014). Therefore, the genetic diversity of HIV-1 in Jiangsu might be underestimated, and some new inter-subtype recombinants might exist but not be identified. Furthermore, the molecular epidemiological characterizations of HIV-1 in some cities of Jiangsu are still unavailable now.

**Fig. 1:**
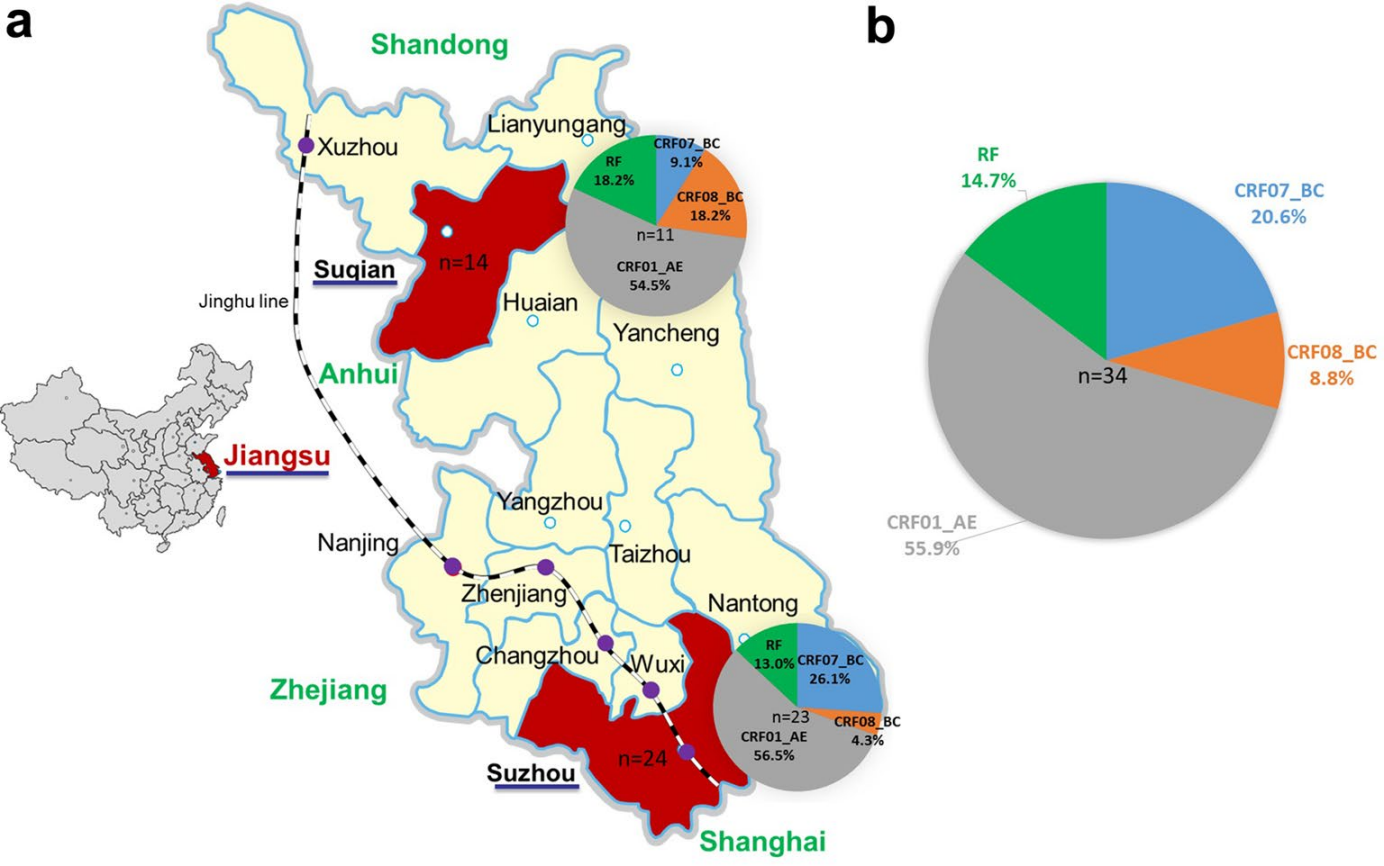
The geographic distribution and subtypes distribution in Suzhou and Suqian. **a** Jiangsu Province, located in the East China, the geographical distribution in China was shown in the *left part* of the (**a**). The provinces, Shandong, Anhui, Shanghai and Zhejiang, adjoining with Jiangsu were shown. The geographical and HIV-1 subtypes distribution of Suzhou and Suqian in Jiangsu Province were shown in the *right part* of the (**a**). Moreover, stations of Jinghu high speed railway within Jiangsu Province were labeled by *purple circle*. *RF* recombination forms. **b** The proportion of HIV-1 subtypes in Jiangsu

In this study, we used multiple genomic fragments (*p17*, *pol*, *vif*-*env* and *C2V3*) of HIV-1 to determine the subtype characterization of HIV-1 circulating in two cities (Suzhou and Suqian) of Jiangsu (Fig. 1a). We found that multiple HIV-1 subtypes, including CRF07_BC, CRF08_BC, CRF01_AE and some inter-subtype recombinants were circulating in the two sampled cities. Our findings provided new molecular epidemiological information for the prevention and control of HIV-1 in Jiangsu and even East China.

## Methods

### Samples, HIV-1 RNA extraction and gene fragments amplification

A total of 38 confirmed HIV sero-positive samples from out-patients and hospitalized patients which were collected from the Second Affiliated Hospital of Soochow University and Suqian CDC. All collected plasma were prepared and stored in a −80 °C freezer until use according to standard procedures. Viral RNAs were extracted from 200 μl of plasma with the MiniBEST Viral RNA/DNA Extraction Kit Ver4.0 (TaKaRa Biotechnology Co. Ltd., Dalian, China) and then subjected to the amplification of five HIV-1 genomic fragments (*p17*, *pol*, *vif*-*vpr*, *vpr*-*env*, *C2V3*). Their locations in HXB2 are 683–1255, 2147–3462, 5084–5805, 5653–6454, and 6817–7381 nt, for *p17*, *pol*, *vif*-*vpr*, *vpr*-*env*, *C2V3*, respectively. Reverse transcription PCR reactions were performed using One-step RNA PCR kit (TaKaRa Biotechnology, Dalian, China). The PCR products were subjected to second PCR (nested PCR) using ExTaq (TaKaRa Biotechnology, Dalian, China). The primer pairs used in this study and the amplification fragments information were consistent with a previous study (Pang et al. 2012). The condition and the procedure of reverse transcription reaction and PCR reactions were performed according to the operation manual. All amplified products were sent to Shanghai Invitrogen Biotechnology Co., LTD. for sequencing.

### HIV genotyping and phylogenetic analyses

HIV reference sequences were downloaded from Los Alamos HIV Sequence Database (www.hiv.lanl.gov). The obtained sequences were aligned together with reference sequences using the Clustal W program implemented in MEGA 5.2 (Tamura et al. 2011), and then manually edited. The phylogenetic trees were constructed using NJ (neighbor-joining) method with a bootstrap evaluation of 1000 replications with MEGA5.2. To determine the potential recombination of HIV-1, the bootscan analyses were performed by SimPlot 3.5.1 software (Lole et al. 1999). The parameters of SimPlot bootscan analysis were as follows: window size, 200 bps; step size, 20 bps; tree algorithm, neighbor; distance model, Kimura; bootstrap replicate, 100; reference type, 50 % consensus.

### GenBank accession numbers

The obtained nucleotide sequences of this study have been submitted to GenBank and are available under the accession numbers of KM054876–KM054975‏.

## Results

### Social-demographic characterization of HIV-1 infected individuals in two cities (Suzhou and Suqian) of Jiangsu

A total of 38 HIV-infected individuals, including 24 (63.2 %) from Suzhou and 14 (36.8 %) from Suqian, were covered in this study. The social-demographic information, including gender, marital status, educational level, age, ethnic, occupation, risk behaviors, is listed in Table 1. Majority of these individuals are male (84.2 %) and ethnic Han (94.7 %). Their marriage status showed that 57.9 % (22/38) were married, and the others were unmarried, divorced or without this information. More than half (55.3 %) of them were 31–40 years old and 44.7 % were unemployed. Sexual contacts, including heterosexual (50.0 %) and homosexual (31.6 %) contacts, were the major risk behavior for HIV-1 infection, accounting for 81.6 % of total. Injecting drug use (IDU) was the second most common factor associated with HIV infection, obviously different from the observations in southwestern (e.g., Yunnan and Guangxi) and northwestern (e.g., Xinjiang) China (Zhang et al. 2002; Liu et al. 2006, 2008; Sun et al. 2011; Yan et al. 2006).

**Table 1:** Social-demographic characteristics of HIV-1 infected Suzhou and Suqian

Variable	Suzhou	Suqian	Count
Location	24	14	38
Gender			
Male	22	10	32 (84.2)
Female	2	4	6 (15.8)
Marital status			
Unmarried	7	3	10 (26.3 %)
Married	14	8	22 (57.9 %)
Divorced	3	2	5 (13.2 %)
Not clear	0	1	1 (2.6 %)
Education level			
≤Primary school	1	4	5 (13.2 %)
Middle school	8	6	14 (36.8 %)
≥High school	15	4	19 (50.0 %)
Age (years old)			
15–20	1	0	1 (2.63 %)
21–30	9	0	9 (23.68 %)
31–40	10	11	21 (55.26 %)
41–50	3	3	6 (15.79 %)
51–60	1	0	1 (2.63 %)
Nationality			
Han	23	13	36 (94.74 %)
Dai	0	1	1 (2.6 %)
Hani	1	0	1 (2.6 %)
Occupation			
Farmer	0	3	3 (7.9 %)
Worker	5	3	8 (21.1 %)
Businessman	3	1	4 (10.5 %)
Unemployment	13	4	17 (44.7 %)
Servicer	3	3	6 (15.8 %)
Mode of contracted HIV			
Homosexual contact	6	6	12 (31.6 %)
Heterosexual contact	14	5	19 (50.0 %)
Injection drug use	3	1	4 (10.5 %)
Uncertain	1	2	3 (7.9 %)
Dual sex partner			
Yes	0	3	3 (7.89 %)
No	24	11	35 (92.11 %)

There are several light differences in social-demographic characterizations between Suzhou and Suqian although the little differences between two cities might be a result of a bias due to small sample size. Most HIV-infected individuals (62.5 %) in Suzhou had completed their high school education, higher than those (28.6 %) in Suqian. Heterosexual contact (58.3 %) was the likely predominant risk factor for HIV infection in Suzhou, while homosexual contact (46.2 %) was the major factor for HIV infection in Suqian. In addition, three individuals (21.4 %) in Suquan self-reported to have multiple sex partners, while no one was in Suzhou.

### The amplification of HIV genomic fragments

From 38 HIV-positive samples, 34 (89.5 %) *p17*, 32 (84.2 %) *pol*, 35 (92.1 %) *vif*-*vpr*, 32 (84.2 %) *vpr*-*env* and 34 (89.5 %) *C2V3* fragments were successfully amplified and sequenced (Table 2). The failure in the amplification of viral genome fragments may be due to primer specificity and low viral load in some specimens. As described in the previous study (Pang et al. 2012), the *vif*-*vpr* (5084–5805 nt in HXB2) fragment overlapped with the *vpr*-*env* (5653–6454 nt in HXB2) fragment, we merged both two fragments into a *vif*-*env* sequence if both sequences available for the same sample. Finally, 27 merged *vif*-*env* sequences were obtained (Table 2).

**Table 2:** Subtype characterizations of four genomic fragments from HIV-infected population in Suzhou and Suqian, Jiangsu

Specimen	*p17*	*pol*	*vif*-*env*	*c2v3*	Total^a^
11jssq001	C	CRF08_BC	NA	C	CRF08_BC
11jssqHS002	CRF01_AE	CRF01_AE	CRF01_AE	NA	CRF01_AE
11jssqHS013	CRF01_AE	CRF01_AE	NA	CRF01_AE	CRF01_AE
11jssqIDU011	NA	NA	CRF07_BC	B/C	B/C
11jssqMSM003	CRF01_AE	CRF01_AE	CRF01_AE	CRF01_AE	CRF01_AE
11jssqMSM004	CRF01_AE	CRF01_AE	CRF01_AE	CRF01_AE	CRF01_AE
11jssqMSM006	C	CRF08_BC	NA	NA	CRF08_BC
11jssqMSM007	CRF01_AE	CRF01_AE	CRF01_AE	CRF01_AE	CRF01_AE
11jssqMSM009	CRF01_AE	CRF01_AE	CRF01_AE	CRF01_AE	CRF01_AE
11jssqMSM010	C	CRF07_BC	CRF07_BC	C	CRF07_BC
11jssqMSM014	NA	CRF01_AE/B	NA	CRF01_AE	CRF01_AE/B
12jssz001	C	CRF07_BC	CRF07_BC	C	CRF07_BC
12jsszHS002	CRF01_AE	CRF01_AE	CRF01_AE	CRF01_AE	CRF01_AE
12jsszHS004	CRF01_AE	CRF01_AE	CRF01_AE	CRF01_AE	CRF01_AE
12jsszHS005	C	CRF07_BC	NA	C	CRF07_BC
12jsszHS008	CRF01_AE	CRF01_AE	CRF01_AE	NA	CRF01_AE
12jsszHS011	CRF01_AE	NA	CRF01_AE	CRF01_AE	CRF01_AE
12jsszHS012	CRF01_AE	CRF01_AE	CRF01_AE	CRF01_AE	CRF01_AE
12jsszHS013	C	B/C	CRF07_BC	CRF01_AE	CRF01_AE/B/C
12jsszHS016	C	CRF07_BC	CRF07_BC	C	CRF07_BC
12jsszHS018	CRF01_AE	CRF01_AE	CRF01_AE	CRF01_AE	CRF01_AE
12jsszHS019	CRF01_AE	CRF01_AE	CRF01_AE	CRF01_AE	CRF01_AE
12jsszHS020	CRF01_AE	CRF01_AE	CRF01_AE	CRF01_AE	CRF01_AE
12jsszHS021	B	B	B	CRF01_AE	CRF01_AE/B
12jsszHS022	C	CRF08_BC	C	C	CRF08_BC
12jsszHS023	CRF01_AE	CRF01_AE	CRF01_AE/B	CRF01_AE	CRF01_AE/B
12jsszHS024	C	CRF07_BC	CRF07_BC	C	CRF07_BC
12jsszMSM003	CRF01_AE	CRF01_AE	CRF01_AE	CRF01_AE	CRF01_AE
12jsszMSM006	C	CRF07_BC	CRF07_BC	C	CRF07_BC
12jsszMSM009	CRF01_AE	CRF01_AE	NA	CRF01_AE	CRF01_AE
12jsszMSM014	C	CRF07_BC	NA	C	CRF07_BC
12jsszMSM015	CRF01_AE	CRF01_AE	CRF01_AE	CRF01_AE	CRF01_AE
13jsszIDU017	CRF01_AE	CRF01_AE	CRF01_AE	CRF01_AE	CRF01_AE
13jsszMSM007	CRF01_AE	CRF01_AE	CRF01_AE	CRF01_AE	CRF01_AE
Total	32	32	27	31	34

If no sequences are available on *pol* and *vif*-*env*, the strains were not taken into account in the statistics

*NA* not available

^a^CRF07_BC and CRF08_BC originated by insertion of several short segments of subtype B into the backbone of subtype C. Both CRFs_BC have genomic segments of subtype C origin in p17 and C2V3 region. Therefore, it is unable to distinguish CRF07_BC/CRF08_BC from subtype C in the phylogenetic trees of *p17* and *C2V3*. Therefore, the genomic segments (i.e. *pol* and *vif*-*env*) including recombination breakpoints are used as major determinants for identification of CRF07_BC or CRF08_BC

### Subtyping of HIV-1 based on four HIV-1 genomic fragments

**Fig. 2:**
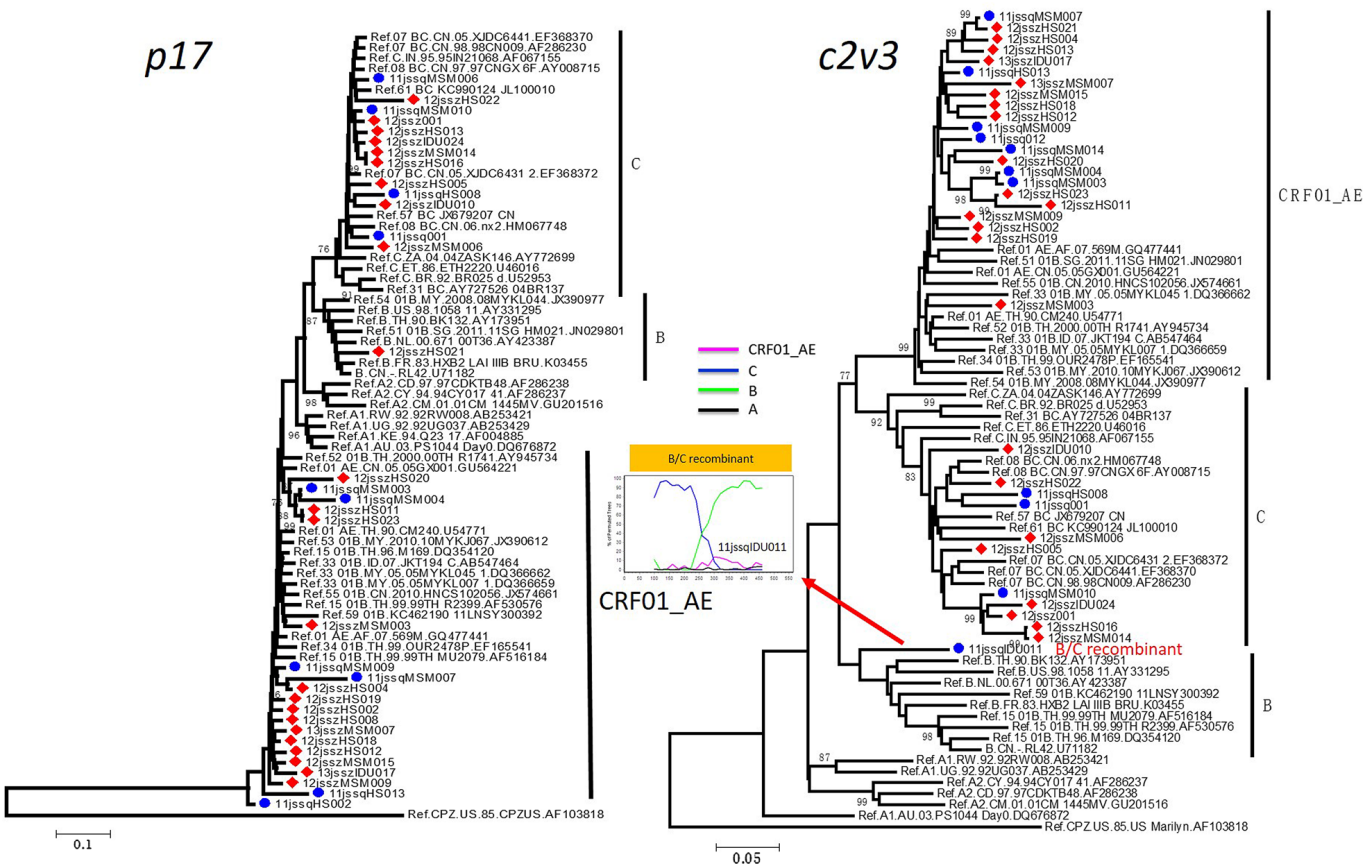
Phylogenetic tree of *p17*, *C2V3* fragments. The subtype references were download from Los Alamos HIV Sequence Database. Notable, the RL42 (U71182) and new CRFs include CRF51 to CRF61 were obtained. The stability of the nodes was assessed by bootstrap analyses with 1000 replications and only bootstrap values of more than 75 are shown at the corresponding nodes. The bootscanning plot of *C2V3* fragment of HIV-1 intersubtype recombinant was shown in the right part

**Fig. 3:**
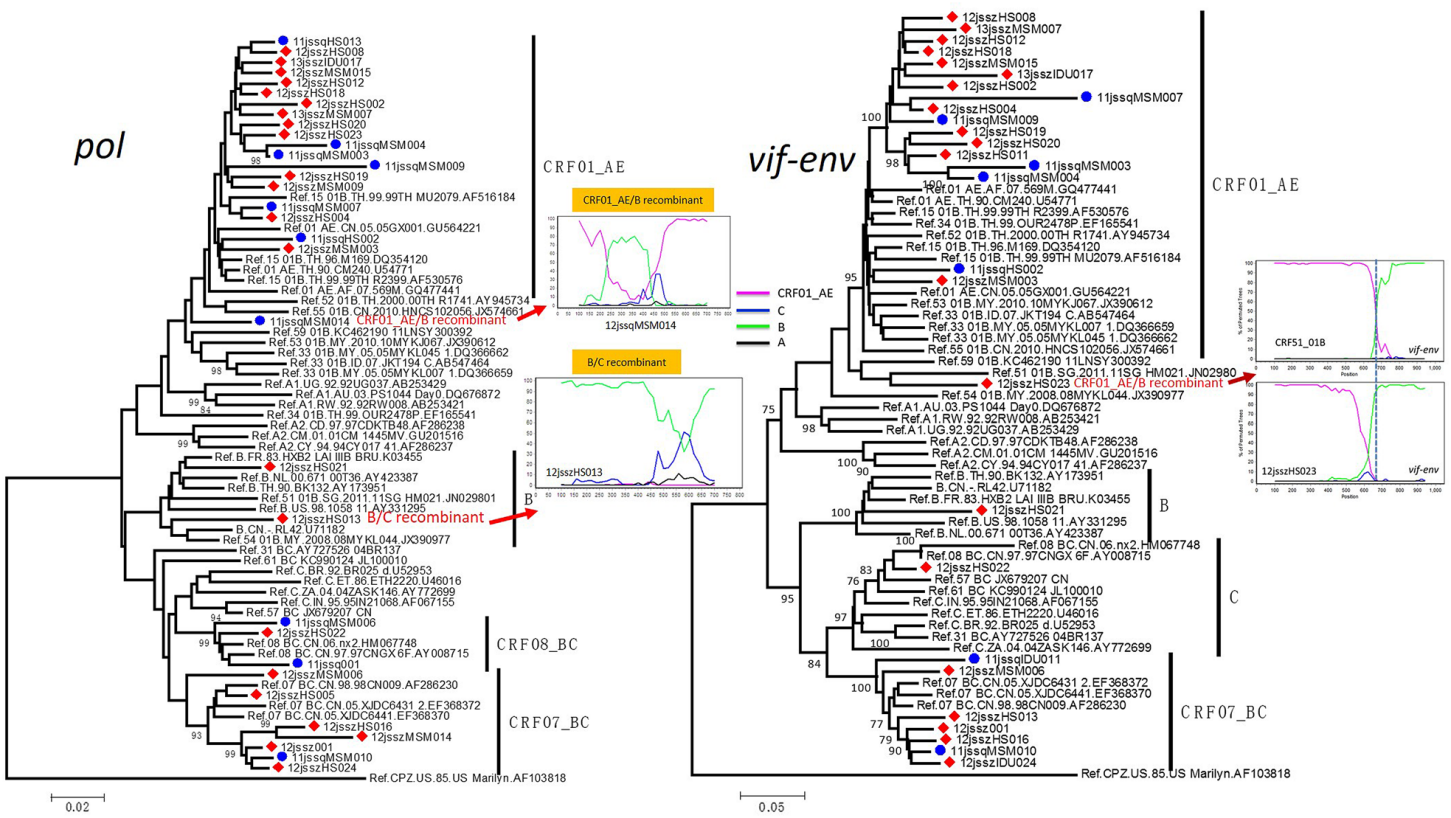
The phylogenetic tree of *pol* and *vif*-*env* fragments of HIV-1 strains isolated from Suqian and Suzhou was constructed with MEGA 5.0 using the neighbor-joining method. The subtype references were download from Los Alamos HIV Sequence Database. A China subtype B strain RL42 (U71182) was also used as a subtype reference. The stability of the nodes was assessed by bootstrap analyses with 1000 replications and only bootstrap values of more than 75 are shown at the corresponding nodes. The bootscanning plots of *pol* and *vif*-*env* fragments of HIV-1 intersubtype recombinants were shown in the each part. There was one BC recombinant to be embedded within the clade of subtype B. The main reason is that the recombinant comprises a genetic backbone of subtype B with a short insertion of subtype C. The too short insertion with few effective information sites from subtype C did not chance the phylogeny of the recombinant

To investigate the subtype characterization of HIV-1 in Suzhou and Suqian, four phylogenetic trees were constructed based on *p17*, *pol*, *vif*-*env* and *C2V3* fragments. Among 34 *p17* sequences, 1 (2.9 %), 13 (38.2 %) and 20 (58.8 %) were identified to be HIV-1 group M subtype B, C and CRF01_AE, respectively (Fig. 2). In *C2V3* tree, except one sequence (11jssqIDU011) that clusters outside the subtype B clade, 11 (32.4 %) and 22 (64.7 %) were clearly identified as HIV-1 group M subtype C and CRF01_AE, respectively (Fig. 3). The strain outside the clades of known subtypes might represent a new inter-subtype recombinant. To determine whether 11jssqIDU011 is a recombinant, the bootscan analysis was performed using Simplot software. The result revealed that 11jssqIDU011 was a B/C recombinant (Fig. 2).

HIV-1 *pol* and *vif*-*env* fragments are the crucial regions for the confirmation of HIV-1 group M genotype, especially for the finding of new recombinant. So, the *pol* and *vif*-*env* sequences from Suzhou and Suqian were also analyzed. The phylogenetic tree of *pol* showed that one sequence (11jssqMSM014) was unable to cluster within the clades of known subtypes (Fig. 3), implying that it might be a recombinant. The bootscan analysis confirmed it being CRF01_AE/B recombinant. To avoid the omission of some recombinants, all other sequences were also subjected to the bootscan analysis in despite that they well clustered within the clades of known subtypes/CRFs in the phylogenetic tree. The bootscan analysis showed that another sequence clustering within the subtype B clade was a B/C recombinant (Fig. 3). As a result, 1 (3.1 %) subtype B, 7 (21.9 %) CRF07_BC, 3 (9.4 %) CRF08_BC, 19 (59.4 %) CRF01_AE and 2 (6.3 %) inter-subtype recombinants were identified based on 32 *pol* sequences (Table 2). In the tree of *vif*-*env*, all sequences clustered within the known subtype/CRF clades. Interestingly, we found that a sequence from Suzhou clustered with a CRF51_01B sequence. CRF51_01B was firstly identified in Singapore, and mainly prevalent here (Ng et al. 2012). To confirm whether it was CRF51_01B, further bootscan analysis was performed. The result showed that this sequence had different recombination breakpoint with CRF51_01B (Fig. 3), indicating that it was a new CRF01/B recombinant, rather than CRF51_01B. As a result, 1 (3.7 %) B, 1 (3.7 %) C, 7 (25.9 %) CRF07_BC, 17 (63.0 %) CRF01_AE and 1 (3.7 %) new recombinant were identified based on *vif*-*env* sequences (Table 2).

### HIV-1 group M subtype characterization in Suzhou and Suqian

To determine genotype of each sample, the subtyping results of four fragments were taken into account together (Table 2). To assure the accuracy, only the samples with one available *pol* or *vif*-*env* sequence were included. According to this standard, four samples (11jssqHS005, 11jssq012, 11jssqHS008 and 12jsszIDU010) that lack both *pol* and *vif*-*env* sequences were excluded in the statistics. Among the remaining 34 samples, 7 (20.6 %) CRF07_BC, 3 (8.8 %) CRF08_BC, 19 (55.9 %) CRF01_AE, and 5 (14.7 %) inter-subtype recombinants were found in two cities Suzhou and Suqian (Fig. 1b). No pure B, B′ and C subtypes were identified. Five recombinants contain one B/C, three CRF01/B and one CRF01/B/C recombinants (Table 2). Because of having different recombination breakpoints, they represent five unique recombinant forms (URFs). These results indicated that multiple HIV-1 subtypes, including CRF01_AE, CRF07_BC, CRF08_BC and some URFs were circulating in Jiangsu, and the predominant HIV-1 genotype was CRF01_AE, followed by CRF07_BC and CRF08_BC (Fig. 1b).

In Suqian, 6 (54.5 %) CRF01_AE, 1 (9.1 %) CRF07_BC, 2 (18.2 %) CRF08_BC, and 2 (18.2 %) URFs were identified (Fig. 1a). In Suzhou, 13 (56.5 %) CRF01_AE, 7 (26.1 %) CRF07_BC, 1 (4.3 %) CRF08_BC, and 3 (13.0 %) URFs were identified (Fig. 1a). Although there is a long geographical distance between Suqian and Suzhou, there was no obvious difference in HIV-1 group M subtype distribution between the two cities (Fig. 1). In particular, CRF01_AE appeared to be the most predominant HIV-1 strains circulating in both cities (Fig. 1a), consistent with the increasing trend of CRF01_AE prevalence in China (Zhang et al. 2014).

In China, CRF01_AE was the most predominant strains circulating in the sexual transmission group (including heterosexuals and homosexuals), and CRF07_BC and CRF08_BC were major circulating among injection drug users (IDUs) (Yang et al. 2002; Su et al. 2000; Piyasirisilp et al. 2000). We investigated the potential association between HIV-1 genotypes and the risk behaviors. In the heterosexuals, the most predominant strains were CRF01_AE (58.8 %), followed by CRF07_BC (23.5 %). Similarly, in the homosexuals, the most predominant stains were also CRF01_AE (61.5 %), followed by CRF07_BC (23.1 %). In addition, among the only two IDUs, one was CRF01_AE and another was B/C recombinant. The emergences of relatively high proportions of CRF07_BC in the sexual transmission group and of CRF01_AE among IDUs might suggest an increasing trend of HIV-1 transmission between different risk groups.

### Comparison of HIV-1 group M subtype distributions between Jiangsu and the surrounding provinces

**Fig. 4:**
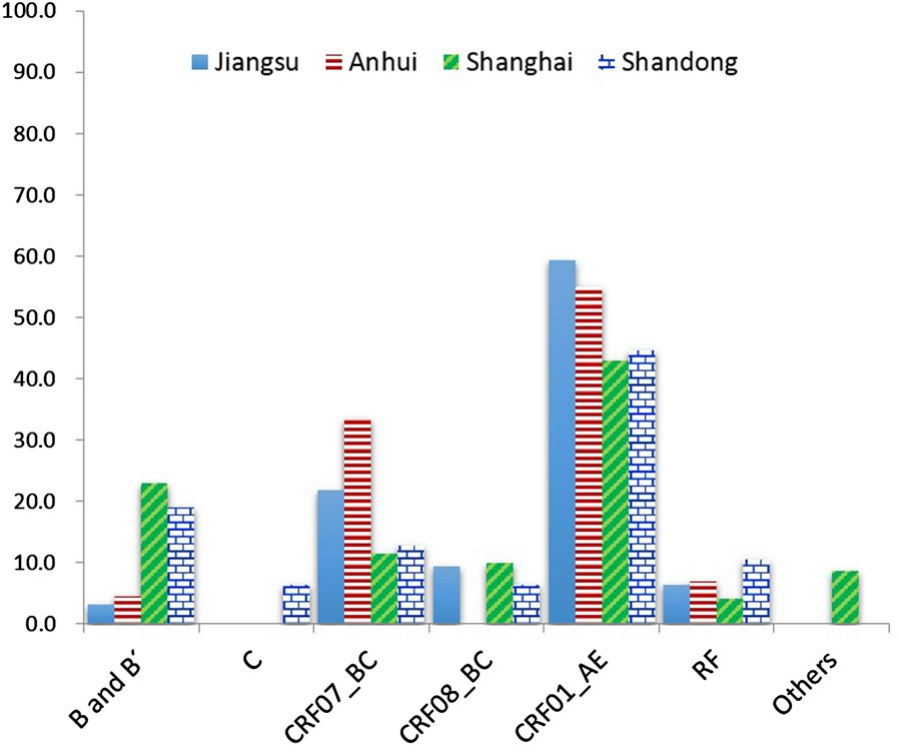
Comparison of HIV-1 subtypes between Jiangsu and surrounding regions. Comparison of HIV-1 subtypes between Jiangsu and surround regions based on *pol* fragment. The data of Shanghai, Anhui and Shandong were retrieved from Wu et al. (2013), Zhong et al. (2007), Zhang et al. (2010)

We further compared the HIV-1 group M subtype distribution between Jiangsu and the surrounding provinces. Because of no data available from Zhejiang, only the data from Shanghai, Shandong, and Anhui were covered. As the subtype results in previous studies were based on the *pol* sequences, comparison was performed only based on the results from *pol* sequences (Fig. 4). The comparison showed that Jiangsu had similar HIV-1 group M subtype distribution to the surrounding provinces. CRF01_AE was the most predominant strains in all four provinces with prevalence of 42.9–59.4 %. The second most common subtype was different in the four provinces with CRF07_BC in both Jiangsu and Anhui and subtype B in both Shanghai and Shandong. HIV-1 inter-subtype recombinants were identified in all four provinces with proportions of 4.2–10.6 %, and no or less pure subtype C strain was found there (Fig. 4). Because only *pol* region was used in HIV-1 genotyping, the actual numbers of HIV-1 inter-subtype recombinants in the four provinces (e.g., east China) might be greater than reported data. In contrast, the numbers of subtypes B and C might be lower than reported data. Most recombinants in these regions were CRF01_AE-associated recombinants, different from the observation in southwestern China, where B/C recombinants were the most common recombinant forms (Pang et al. 2012).

## Discussion

IDU and former commercial plasma donation were the two major modes of HIV transmission in China during 1985 to 2005, accounting for 44.2 and 29.6 % of total infections, respectively (Ministry of Health of the People’s Republic of China, Joint United Nations Programme on HIV/AIDS, World Health Organization 2012; Yan et al. 2006; Zhang et al. 2004). In recent years, the number of new HIV-1 infections caused by sexual contacts, including heterosexual and homosexual contacts, had increased rapidly and sexual transmission had become the primary mode of HIV infection in China (Ministry of Health of the People’s Republic of China, Joint United Nations Programme on HIV/AIDS, World Health Organization 2012; Guo et al. 2009a; Wu et al. 2013; Ye et al. 2014; An et al. 2012; Xu et al. 2013). Among the 48,000 new infections in China in 2011, approximately 81.6 % was associated with sexual exposure (Ministry of Health of the People’s Republic of China, Joint United Nations Programme on HIV/AIDS, World Health Organization 2012). The similar trend could be also observed in Jiangsu. The investigation conducted in 2011 showed that 87.26 % of PLHIV in Jiangsu acquired HIV-1 infection via sexual contact, including 46.6 % heterosexual and 40.64 % homosexual contacts (Control JPCfDPA 2012). Our results showed that 81.6 % (heterosexuals: 50.0 % and homosexual: 31.6 %) of HIV infected cases in Suzhou and Suqian acquired infections through sexual contacts, very close to the data of whole province. These imply that the prevention and control of HIV-1 should focus on sexual transmission group in Jiangsu. One difference between Suzhou and Suqian was that more HIV-infected individuals (62.5 %) in Suzhou have higher education level than in Suqian (28.6 %), which might be attributed to higher economic development and cultural education levels in Suzhou than in Suqian.

Jiangsu is one of the most developed areas in China (Fig. 1a), and serves as a crucial transportation hub linking Shanghai and other regions of China. Therefore, there are a large number of migrant populations from some undeveloped areas to work and live in Jiangsu or go through Jiangsu to Shanghai or Zhenjiang, another two most developed provinces in China, which may directly or indirectly facilitate the spread of and co-circulation of various human viruses or subtypes of certain virus in this area. Previous report showed that about 72.0 % of HIV-positive persons in Jiangsu were living in Nanjing, Suzhou, Wuxi, Changzhou and Xuzhou cities (Control JPCfDPA 2012), all which are located in the important lines (including railway, high-speed railway and high-speed way)crossing Jiangsu. These may support a potential association between a large scale of population mobility and HIV transmission.

Previous results based on *gag* and *C2V3* fragments showed that seven HIV-1 group M subtypes, including B, C, CRF01_AE, CRF02_AG, CRF07_BC, CRF08_BC, and A1 were circulating in Jiangsu (Yang et al. 2009). The co-circulation of multiple HIV-1 group M subtypes will increase the chance to generate new HIV-1 recombinant (Guo et al. 2014). In this study, we detected five (14.7 %) recombinants from 34 samples collected in Suzhou and Suqian. Apart from these recombinants, we also detected 19 (55.9 %) CRF01_AE, 7 (20.6 %) CRF07_BC, 3 (8.8 %) CRF08_BC, but no pure subtype B and C, showing an obviously different genotype distribution of HIV-1 group M with previous ones (Li et al. 2013). These possibly suggest a new molecular epidemiological trend of HIV-1 in Jiangsu.

HIV-1 genotype distribution in Jiangsu (including Suzhou and Suqian) was similar to those in the surrounding provinces (e.g., Shanghai, Anhui, and Shandong) (Wu et al. 2013; Zhong et al. 2007; Zhang et al. 2010). CRF01_AE appeared to be the most predominant HIV-1 genotypes in the four provinces (Fig. 4). Among five recombinants identified in Jiangsu, four (80 %) were CRF01_AE-involoved recombinants, including three CRF01/B and one CRF01/B/C recombinants, consistent with the observation in the three surrounding provinces, where 68.8 % (11/16) of recombinants were CRF01_AE related (Wu et al. 2013; Zhong et al. 2007; Zhang et al. 2010). High proportion of CRF01_AE-involved recombinants might be the result of high prevalence of CRF01_AE in the East China. In addition, only one B/C recombinant was identified in Suzhou and Suqian. Interestingly, no pure HIV-1 group M subtypes B and C strains were found. It implies that there may be two reasons for the origin of these subtype B and C-involved recombinants. First, the B and C fragments of these recombinants might come from CRF07_BC or CRF08_BC. Second, these recombinants might originate in other regions where B and C subtypes were circulating and be introduced into Suzhou and Suqian. Furthermore, no finding of pure HIV-1 group M subtype B and C strains also implies that fewer B/C recombinants will be generated in Jiangsu (at least in Suzhou and Suqian) in future. On the other hand, CRF01_AE and CRF07_BC were the top two predominant HIV-1 group M subtypes in Jiangsu and Anhui (Fig. 4), which implies that more CRF01/CRF07 recombinants will be generated in the two provinces in future. In fact, two CRF01/CRF07 recombinants had been identified from one IDUs in Jiangsu and three MSM in Anhui previously (Wu et al. 2013; Guo et al. 2009b). Therefore, to effectively monitoring the changes of HIV-1 diversity in this area, a series of large-scale molecular epidemiological investigation are needed.

## Conclusion

In this study, by characterizing the subtype distribution of HIV-1 group M in Suzhou and Suqian, we found that CRF01_AE was the most predominant HIV-1 group M subtypes in Jiangsu, and less and/or no pure subtype B and C was currently circulating here. We predicted that more CRF01/CRF07 recombinants, but fewer B/C recombinants will be generated in Jiangsu in future. Additional, we also suggest that the actual number of HIV-1 inter-subtype recombinants is larger and the genetic diversity of HIV-1 group M in Jiangsu is more complex than described here. Therefore, a molecular epidemiological investigation based on all high-risk groups in whole Jiangsu province is needed for providing detailed information for the prevention and control of HIV-1 in Jiangsu and even whole east China.
